# Nursing Students’ Perceptions of Factors Influencing Nursing Intentions toward COVID-19 Patients

**DOI:** 10.3390/healthcare12030285

**Published:** 2024-01-23

**Authors:** Nari Lee, Hae Ran Kim

**Affiliations:** 1 Chosun University Hospital, Gwangju 61453, Republic of Korea; nari6318@hanmail.net; 2 Department of Nursing, College of Medicine, Chosun University, Gwangju 61452, Republic of Korea

**Keywords:** COVID-19, intention, nursing, students, patients

## Abstract

Coronavirus disease (COVID-19) is a pandemic to which nursing students are particularly susceptible. This study aims to comprehensively examine nursing students’ knowledge, attitudes, risk perceptions, preventive behaviors related to COVID-19, and nursing intentions toward patients with the virus. A questionnaire was administered to 149 nursing students from two universities. Data on the respondents’ general characteristics, knowledge levels, attitudes, perceived risk, preventive behaviors toward COVID-19, and nursing intentions toward COVID-19 patients were collected. The collected data were statistically analyzed using SPSS software (version 26.0). This involved descriptive statistics, independent *t*-tests, one-way ANOVA, Pearson’s correlation coefficient, and stepwise multiple regression analyses. The analyses of the factors affecting nursing students’ nursing intentions for COVID-19 patients showed that the most predictive factor was perceived risk (β = −0.38, *p* < 0.001), followed by attitudes (β = 0.29, *p* < 0.001) and preventive behaviors (β = 0.17, *p* = 0.017), which explained 26% of the variance in nursing intentions. Lowering the perceived risk of infectious diseases and cultivating positive attitudes and preventive behaviors can increase nursing students’ intentions toward COVID-19 patients. Finally, infection management education programs and research on interventions for nursing students are necessary to enhance the quality of nursing care provided to patients with novel infectious diseases.

## 1. Introduction

Coronavirus disease (COVID-19) emerged as a major global challenge in 2020, with limited information on epidemiologic evidence and no clinically proven vaccine or treatment [1]. At the time, governments responded proactively by building healthcare facilities, deploying healthcare professionals to contain the spread, and expanding treatment capacity, as seen in the efforts of the Korea Centers for Disease Control and Prevention [KDCA] [2]. At the national policy level, healthcare engagement became increasingly important, complementing disease response and surveillance efforts.

As we enter 2024, it is appropriate to recognize the evolution of knowledge surrounding COVID-19. Significant progress has been made in our understanding of the virus, as well as in vaccine development and treatment strategies. Due to the fact that 2020 was a time of uncertainty characterized by limited knowledge about the virus, several studies including this one were initiated to contribute to our collective understanding. The data collected from nurses in 2020 are valuable, providing insight into the challenges they faced during this critical time in the pandemic.

Nurses are important members of the medical teams that responded to COVID-19. Their willingness to actively participate in treating infected patients is important for the effective management of novel infectious diseases [3]. According to a 2021 study, more than 1000 nurses from 50 countries died from COVID-19 [4]. Despite awareness of the risks to individual healthcare workers in the context of the pandemic, several medical professionals were willing to stay with infected patients and provide them with care [5,6].

Nursing intentions indicate nurses’ voluntary and proactive nursing behaviors. Confirming nurses’ willingness to provide specialized nursing care independently during a pandemic is crucial for improving nursing quality [7]. The lack of clear information, especially at the beginning of the pandemic, on the precise mechanisms, transmission routes, and symptoms of novel infectious diseases hampers nursing intentions due to conflicting responsibilities and roles. Indeed, nurses frequently exposed to SARS-CoV-2 experience a fear of infecting themselves and their families [8,9], psychological distress, and high levels of fatigue resulting from long and intense working hours [10]. Moreover, when nurses witness colleagues contracting novel infectious diseases, they are more likely to avoid situations involving such patients [11,12]. Nurses provide direct nursing care to suspected and confirmed patients 24 h a day in various healthcare settings such as isolation wards, intensive care units, surgical wards, and screening clinics. Thus, they are at high risk of contracting infectious diseases [13], increasing the likelihood of transmitting illnesses to other patients, colleagues, and the local community [14]. In particular, some categories of patients already predisposed to the risk of infection, such as surgical patients, may be more susceptible to the emergence of additional infectious problems such as SARS-CoV-2 [15]. These considerations underline nurses’ responsibility to promote an effective safety culture and the importance of being predisposed to infectious diseases to reduce their negative consequences. Therefore, it is important to reduce the negative impact of infectious diseases on nurses and promote the positive aspects of nursing intentions. So doing may increase the likelihood of evidence-based nursing interventions and the overall effectiveness of nursing practice. 

Unlike other illnesses, novel infectious diseases can spread rapidly, have a high level of contagion, overwhelm healthcare systems, and cause several disruptions. Future nurses will have to provide care to patients with novel infectious diseases, and those with low nursing intentions will burden the response of the healthcare system, for example, through a shortage of healthcare personnel in hospitals during outbreaks. Indeed, after the COVID-19 pandemic, nursing students in clinical practice have experienced anxiety and fear when providing nursing care [8,9], and these negative effects increased the likelihood of them avoiding nursing care. Studies have emphasized the importance of assessing nursing students’ willingness to provide nursing care to infected patients in situations involving novel infectious diseases as a direct factor that influences the quality of nursing care [16]. Hung, Lam, Chow, Ng, and Pau (2021) also emphasized the importance of assessing nursing students’ willingness to provide nursing care for these patients. They found that nursing students with a higher level of knowledge about novel infectious diseases displayed a greater willingness to provide care [17]. Despite the significance of investigating nursing students’ willingness to care for COVID-19 patients, there remains a shortage of related research. Therefore, understanding their willingness to care for COVID-19 patients is important for enhancing their future competence in caring for patients with novel infectious diseases and improving the quality of nursing care.

The knowledge, attitude, and practice (KAP) model is a health education theoretical framework that proposes the modification of health behaviors through the acquisition of knowledge, formation of beliefs, and development of behaviors. Nursing students’ knowledge and information can influence their attitudes toward COVID-19 and, ultimately, their preventive behaviors [18]. Studies have found that healthcare students with knowledge and positive attitudes were more likely to engage in preventive behaviors during the COVID-19 pandemic [19], and that nursing students’ higher levels of risk perception regarding COVID-19 were associated with a higher degree of adherence to infection prevention measures [20]. A study targeting nursing students with Middle East Respiratory Syndrome (MERS), a novel infectious disease, found that students with higher levels of knowledge about MERS and positive attitudes had higher rates of adherence to preventive behaviors [21]. The same study found that a heightened risk perception of MERS was associated with a higher rate of adherence to preventive behaviors. Furthermore, it is essential to assess nursing students’ knowledge, attitudes, and risk perceptions regarding COVID-19 [22]. Research on these aspects as well as nursing students’ adequate preventive behaviors and nursing intentions regarding patients with COVID-19 remains insufficient. During this crisis, it is crucial to plan effective educational interventions for COVID-19 and ensure the delivery of high-quality nursing care to patients.

Nursing students are healthcare professionals who will have to perform their roles and duties even when new infectious diseases emerge. Due to the fact that voluntary nursing performance is a direct factor in determining the quality of nursing, it is essential, as mentioned, to understand nursing students’ intentions to care for patients with novel infectious diseases [23,24]. 

The year 2020 was particularly critical, as the world grappled with the onset of the COVID-19 pandemic, raising awareness of the challenges facing healthcare professionals. This study recognizes the evolving healthcare landscape and nursing students’ continued commitment to the profession. It aims to explore nursing students’ intentions to care for patients with COVID-19, a topic currently underrepresented in the literature. The term “current” is revisited in this context to acknowledge that the study began in 2020 and to avoid the implications of a real-time assessment. Therefore, this study seeks to assess nursing students’ knowledge, attitudes, risk perceptions, preventive behaviors for COVID-19, and intentions to care for patients with the virus. It also seeks to identify factors that shape these nursing intentions. By clarifying nursing students’ nursing intentions in the context of COVID-19, this study provides important insights for the development of educational programs. 

In summary, to address the gaps in the literature, the objectives of this study are to evaluate participants’ knowledge, attitudes, risk perceptions, preventive behaviors, and nursing intentions related to COVID-19; investigate nursing intentions toward COVID-19 patients based on participants’ general characteristics; examine correlations among participants’ knowledge, attitudes, risk perceptions, preventive behaviors, and nursing intentions; and identify key factors influencing nursing intentions for COVID-19 patients.

## 2. Methods

### 2.1. Design and Participants

This study used a cross-sectional descriptive research design. The participants were nursing students attending a four-year course at a university in G City, Republic of Korea, who were selected using convenience sampling. Data were collected between October and December 2020. The sample size was determined using the G*power 3.1.9.7 program, considering the minimum number of participants required for the multiple regression analysis. With a significance level (α) of 0.05, power (1-β) of 90%, and an effect size of 0.15, and considering 9 variables (4 independent variables and 5 participant characteristics) for the multiple regression analysis, the minimum sample size required was 141 participants. The effect size was determined to be 0.15, considering the scarcity of relevant studies based on prior literature [25,26,27]. Assuming a dropout rate of 10%, 155 questionnaires were distributed, and 96.1% were returned, resulting in a final analysis of 149 questionnaires.

### 2.2. Measures

#### 2.2.1. Knowledge about COVID-19

This study reconfigured the knowledge scale for COVID-19 based on the tool developed by Taghrir et al. [20]. The scale was initially translated into Korean by a professional translator in the medical field and then translated back into English to enhance semantic similarity. This instrument consisted of nine items, and its content validity (CVI) was assessed by five experts: one infectious disease professor, one infectious disease resident, one nursing professor, and two infectious disease nurses with more than ten years of experience. Correct answers scored 1 point, whereas incorrect answers scored 0 points. The total scores were converted into percentiles. Higher scores indicated greater knowledge of COVID-19. The final CVI of the scale was 0.96, and its reliability, as measured by the Kuder–Richardson Formula 20, was 0.51.

#### 2.2.2. Attitudes about COVID-19: Nursing Students’ Trust in Government Guidelines for COVID-19

We reconstructed a tool to assess nursing students’ trust in the government guidelines for COVID-19 based on the work by Zhong et al. [28] and Peng et al. [29]. This tool consists of three items. Its CVI, as rated by five experts, was 0.93. Responses were measured on a five-point Likert scale, and the average score of the three items was calculated. A higher score indicated higher trust in the government guidelines for COVID-19. Cronbach’s α was 0.816.

#### 2.2.3. Perceived Risk of COVID-19 Infection

This study reconstructed a tool to assess the perceived risk of COVID-19 infection based on the work by Taghrir et al. [20] and Rana et al. [30]. This tool consists of three items. Its CVI, as rated by five experts, was 0.93. Responses were measured on a five-point Likert scale, and the average score of the three items was calculated. A higher score indicated a higher perceived risk of COVID-19 infection. Cronbach’s α was 0.577.

#### 2.2.4. Preventive Behaviors against COVID-19

This study reconstructed a tool to assess the preventive behaviors of nursing students against COVID-19 based on Taghrir et al. [20]. This tool consists of 13 items. Its CVI, as rated by five experts, was 0.97. Responses were measured on a 3-point Likert scale (0 = never practiced, 1 = occasionally practiced, and 2 = always practiced), and the average score of the 13 items was calculated. Higher scores indicated higher adherence to preventive behaviors against COVID-19. Cronbach’s alpha was 0.79.

#### 2.2.5. Nursing Intentions

Approval was obtained using a tool developed by Lee [31]. The tool consisted of three items. Responses were measured on a seven-point Likert scale ranging from −3 (not at all true) to +3 (very true). The mean scores for the three items were calculated. A higher score indicated stronger nursing intentions for COVID-19 patients. Cronbach’s α was 0.94.

### 2.3. Procedure

Prior to data collection, we obtained approval from the Institutional Review Board of C University Hospital in Republic of Korea (No. CHOSUN202009001-HE003). Considering the spread of infection during the COVID-19 pandemic, the researchers collected online data. Google Forms was used for the Internet survey, and the estimated time for completion was ten minutes. The survey platform restricted multiple responses to prevent duplication. Upon accessing the survey questionnaire, participants were presented with an initial screen explaining the purpose of the research and an option to indicate their consent to participate. Clicking on this button was considered a voluntary agreement to participate in the study.

### 2.4. Statistical Analysis

Descriptive statistics were used to assess participants’ general characteristics, knowledge of COVID-19, attitudes, perceived infection risk, preventive behaviors, and nursing intentions toward COVID-19 patients. Independent *t*-tests, one-way ANOVA, and Scheffé’s post hoc tests were performed to compare the nursing intentions toward COVID-19 patients based on participants’ general characteristics. Pearson’s correlation coefficient was employed to examine the correlations between knowledge, attitudes, risk perception, preventive behaviors, and nursing intentions related to COVID-19. Finally, a stepwise multiple regression analysis was conducted to identify the factors influencing nursing intentions toward COVID-19 patients. All statistical analyses were performed using SAS version 9.4 software. 

## 3. Results

### 3.1. General Characteristics

Of the participants, 89.3% were female, 33.6% were fourth-year students, 55.7% reported “good” subjective health status, and 43% reported engaging in clinical practice during the COVID-19 pandemic (Table 1).

### 3.2. Nursing Students’ Knowledge about COVID-19, Trust in Government Guidelines, and Risk Perceptions

The participants’ COVID-19 knowledge accuracy rate was 69.8%, with more than 90% of them providing correct answers on transmission methods, hand washing, waste management, and the incubation period. In contrast, lower knowledge accuracy rates were observed for specimen collection methods (26.2%), specimen collection sites (40.9%), initial symptoms (49.7%), and isolation duration (51.7%). Participants’ trust in government guidelines for COVID-19 scored an average of 3.74 ± 0.76 out of 5, with trust in hospital guideline recommendations scoring the highest at 3.98 ± 0.87. The risk perception of COVID-19 averaged 3.98 ± 0.63 out of 5, with the highest score for the risk perception of family transmission infection at 4.42 ± 0.70 (Table 2).

### 3.3. Preventive Behaviors against COVID-19

The average score for COVID-19 preventive behaviors was 1.55 ± 0.29, with the highest scores observed for coughing and hand washing management as well as reducing outdoor shopping. The lowest scores were recorded for reducing the use of public transportation (1.09 ± 0.64) and maintaining hygiene for frequently touched objects (1.09 ± 0.68) (Table 3).

### 3.4. Nursing Intention toward COVID-19 Patients

Nursing intention scored an average of 1.16 ± 1.40 points (−3–+3). While active participation in the nursing care of COVID-19 patients scored the highest at 1.31 ± 1.34 points, voluntary participation in patient nursing care scored the lowest at 0.93 ± 1.64 points (Table 4).

### 3.5. Differences in Nursing Intentions toward COVID-19 Patients Based on General Characteristics

Male students showed higher nursing intentions toward COVID-19 patients (1.81 ± 1.09) than female students (1.08 ± 1.41) (t = 2.011, *p* = 0.046). Those with a “good” subjective health status (1.43 ± 1.16) showed higher nursing intentions than those with a “poor” health status (0.52 ± 1.94) (F = 4.144, *p* = 0.018) (Table 5).

### 3.6. Correlation of Variables

Nursing intentions showed a statistically significant negative correlation with risk perception (r = −0.36, *p* < 0.001) and a significant positive correlation with attitudes (r = 0.34, *p* < 0.001) and preventive behaviors (r = 0.19, *p* = 0.020) (Table 6). Low risk perception, positive attitudes, and high levels of preventive behaviors were associated with higher nursing intentions toward COVID-19 patients.

### 3.7. Factors Influencing Nursing Intentions toward COVID-19 Patients

A stepwise multiple regression analysis was conducted to examine the factors influencing nursing intentions regarding COVID-19 patients. The analysis showed that nursing students’ attitudes, risk perceptions, and preventive behaviors related to COVID-19 were significantly correlated with sex and subjective health status, with significant differences in general characteristics as independent variables. The dependent variable was nursing intentions toward COVID-19 patients. Sex and subjective health status were used as dummy variables. The correlation coefficients of the independent variables were less than 0.80, confirming their independence. Thus, the analysis considered all variables. The case diagnostics showed no outliers, with absolute values greater than 3. To ensure the validity of the linear regression analysis, the basic assumptions of multicollinearity, residuals, and outliers were tested. The Durbin–Watson statistic was used to assess the autocorrelation of the error terms. The result of 2.05, which is close to 2, indicates the absence of autocorrelation. The tolerance values ranged from 0.94 to 0.98, all exceeding the threshold of 0.1. Furthermore, the variance inflation factors (VIFs) ranged from 1.02 to 1.07, well below the threshold of 10, indicating the absence of multicollinearity among the independent variables. A residual analysis was performed to assess the assumptions of normality, linearity, and homoscedasticity of the error terms. Examination of the histograms, normal probability plots (P-P plots), and residual scatterplots revealed a normal data distribution, satisfying the linearity assumption, whereas individual residuals showed homoscedasticity. In addition, an examination of the maximum Cook’s distance to identify outliers showed that the maximum value did not exceed 1, thus confirming the validity of the regression analysis results.

An analysis of the factors influencing participants’ nursing intentions toward COVID-19 patients revealed that the most significant predictor of nursing intentions was risk perception of COVID-19 (β = −0.38, *p* < 0.001), followed by attitudes (β = 0.29, *p* < 0.001) and preventive behaviors (β = 0.18, *p* = 0.017). The adjusted R-squared value, which indicated the explanatory power of the model, was 0.26, indicating that the model explained 26.0% of the variance in nursing intentions. The regression model was statistically significant (F = 17.85, *p* < 0.001) (Table 7).

The following variables were included in the stepwise method: attitudes, perceived risk, and preventive behaviors against COVID-19; sex = dummy variable (male = 1); subjective health status = dummy variable (poor = 1); SE = standard error; and Adj = Adjusted.

## 4. Discussion

This study assessed nursing students’ knowledge, attitudes, risk perceptions, preventive behaviors, and nursing intentions regarding COVID-19 to identify the factors influencing their nursing intentions toward COVID-19 patients. The average knowledge score for COVID-19 in this study was 69.8 points. This score was lower than those reported in previous studies that used similar instruments and items to evaluate COVID-19 knowledge. Previous studies reported an average score of 77.2 points for nursing students [32], 85 points for healthcare students [19], and 86.96 points for medical students [20]. In this study, more than 40% of the participants were first- or second-year students who lacked clinical experience or knowledge. They may find it challenging to respond to difficult questions. Therefore, future studies should develop a questionnaire that considers participants’ level of education. Moreover, early education programs for lower-grade students may improve their knowledge of novel infectious diseases. Furthermore, we propose conducting a comparative study targeting senior students using the developed questionnaire. Regarding individual questions, the correct response rates were high for items related to the transmission of COVID-19, importance of hand washing, management of infectious waste by patients, and incubation period. In contrast, questions on specialized areas frequently encountered in clinical practice, such as specimen collection methods, specimen collection locations, and criteria for isolation release, had lower correct response rates. Nursing students with no experience in caring for COVID-19 patients may have had lower correct response rates to these questions based on their lack of specialized knowledge and experience. Similar results were observed in previous studies conducted on healthcare [19,21,27,33], nursing [34,35,36], and medical [20] students. At the government level, promotion through the media and various channels has enabled the dissemination of information on various infectious diseases, including prevention methods and situational updates. However, there is a deficiency in providing specialized and detailed education on novel infectious diseases to nursing students, who are the future healthcare professionals responsible for the direct nursing care of patients during such disease outbreaks. During outbreaks of novel infectious diseases, media-based learning tends to focus on general preventive guidelines. Therefore, nursing students should receive continuous education and specialized content on novel infectious diseases. 

Nursing students’ trust in the government guidelines scored 3.74 5, which was generally good. This score was similar to that found in previous studies on nursing students’ attitudes toward COVID-19 [23]; healthcare students’ attitudes toward MERS [37]; attitudes of nurses with no experience in nursing emerging respiratory infections [35]; and healthcare students’ attitudes toward COVID-19 [19]. The highest attitude score was observed for hospital guidelines. Nursing students with experience in healthcare facility infection control may believe they can protect themselves from COVID-19 [18]. Therefore, infection control education programs may have a positive impact on attitudes toward novel infectious diseases. The latest research found that prolonged social distancing measures led to increased daily life stress among most university students, potentially contributing to higher social anxiety and leading to lower trust in government policies [38].

The average risk perception score for COVID-19 was 3.98 out of 5, which is lower than that of nursing students (4.45 out of 5) [32]. However, it was higher than the risk perception scores for COVID-19 among medical students (4.08 out of 8) [20]. The highest fear perception was associated with family infections caused by COVID-19. This aligns with previous research findings that individuals often fear infecting their families or those around them when caring for patients with novel infectious diseases [39,40,41].

The average score for COVID-19 preventive behaviors was 1.55 out of 2 points. While direct comparisons are challenging due to differences in measurement tools, this score is relatively higher than that in previous studies on nursing students, where preventive behaviors for COVID-19 [23], preventive behaviors for MERS scored 2.85 out of 5 points [20] and 34.55 out of 48 points [42], and preventive behaviors for MERS among healthcare students scored 2.58 out of 5 points [37]. Large opportunities for exposure to basic infection prevention guidelines and prolonged pandemics may have contributed to the maintenance of personal hygiene. The scores for preventive behaviors in everyday life, such as taking precautions when gathering with friends, using public transportation, and disinfecting surfaces in the surrounding environment, were low, as reported in previous studies [20,21,37]. Thus, nursing students need to be educated on the importance of basic hygiene practices such as hand washing, the preventive effects of lifestyle restrictions, and management of the surrounding environment. Previous studies have found that adherence to standard precautions during clinical practice has a direct impact on infection prevention activities when individuals become healthcare professionals in the future [43]. Furthermore, for COVID-19, the KDCA emphasized the importance of adhering to standards and droplet and airborne precautions. Nursing education should therefore include these aspects to prepare students for novel infectious diseases [44].

Nursing intentions regarding COVID-19 scored 1.16 points, indicating higher-than-moderate nursing intentions. Previous studies assessing the nursing intentions of clinical nurses caring for patients with novel infectious diseases using the same instrument reported the following results. A study conducted before the COVID-19 pandemic by Lee found a nursing intention score of 0.31 points (on a range of −3 to 3) [31], while Moon and Park found a score of 0.17 points [45]. A study assessing the nursing intentions of patients with SARS showed a nursing intention score of 0.56 points [46]. Studies conducted during the COVID-19 pandemic confirmed the following nursing intention scores: Song and Yang a score of 0.57 points [47], and Jeong and Kim a score of 1.04 points [48]. This could be due to the differences in fatality rates and treatment methods based on the type of infectious disease and positive changes in controlling and dealing with novel infectious diseases due to recurring outbreaks. The Republic of Korea has become a prominent model for crisis management among advanced nations, with high levels of support and interest from the government and public, as well as the selfless dedication and competence of COVID-19 healthcare workers [49], contributing to higher nursing intentions. The lowest nursing intention for COVID-19 was found for “voluntarily participating in the care of COVID-19 patients”. Given the adverse impact of COVID-19 on nurses’ mental health, especially among nursing students with relatively limited clinical experience and knowledge, negative influences such as fear of infectious diseases may be even more significant. This underscores the need to develop strategies to reduce the negative impact of infectious diseases on nursing students and enhance their voluntary nursing intentions for patients.

An analysis of nursing intentions toward COVID-19 patients based on participants’ general characteristics revealed that female students had lower nursing intentions than male students [31,39,50,51]. Furthermore, women generally have higher risk perceptions and greater concerns about personal or family infections than do men. However, this study considered a significantly higher proportion of female students than male students, suggesting the need for future comparative studies using samples with a more balanced sex distribution. Furthermore, participants with good subjective health had higher patient care intention scores than those with less favorable health conditions. Positive health status perceptions serve as predictive factors for attitudes and health-promoting behaviors, with positive self-assessments enhancing self-determination and intrinsic motivation [52].

An examination of the correlations between knowledge, attitudes, risk perception, preventive behaviors, and nursing intentions for COVID-19 patients demonstrated that a lower risk perception of COVID-19 was associated with more positive attitudes, and higher performance of preventive behaviors was associated with higher nursing intentions toward COVID-19 patients. In addition, greater knowledge of COVID-19 and more positive attitudes were associated with more preventive behaviors. Studies examining the correlations between nursing students and novel infectious diseases found that higher knowledge levels [21,27,53] and more positive attitudes [27,53] were associated with increased adherence to preventive behaviors. Furthermore, research on healthcare students showed that higher knowledge levels and more positive attitudes were associated with higher compliance with preventive behaviors [19,37].

The examination of the factors influencing nursing intentions for COVID-19 patients indicated that the most significant factor was risk perception of COVID-19, followed by attitudes and preventive behaviors. Lower risk perception, more positive attitudes, and higher preventive behavior compliance were associated with higher nursing intentions toward COVID-19 patients. These results align with the findings of previous research on nursing students, namely that higher knowledge and more positive attitudes were associated with increased compliance with preventive behaviors and higher nursing intentions [23,24], and that higher perceived risk led to lower nursing intentions [25,26,54]. Higher risk perceptions of infection seem to influence anxiety about potential health deterioration due to COVID-19, which could impact nursing intentions toward COVID-19 patients. Park et al. [23,24] suggested that higher anxiety due to novel infectious diseases could lead to increased risk perceptions [23,24]. Therefore, detailed and in-depth education on novel infectious diseases may help reduce infection-related risk perceptions and ultimately contribute to higher nursing intentions.

This study has several limitations. First, this study surveyed nursing students from specific regions regarding their nursing intentions toward COVID-19 patients, making it challenging to generalize the results. Thus, the external validity of the findings should be carefully considered in the context of the broader nursing student population. While this study focused on the vulnerability of nursing students to the COVID-19 pandemic, the generalizability of the findings may be affected by the specific characteristics of the students sampled at the two universities. To increase the external validity of our findings, future studies could include a more geographically and demographically diverse sample of nursing students. In addition, exploring the applicability of the factors identified in different educational and healthcare settings would help in developing a more comprehensive understanding of nursing intentions toward COVID-19 patients in different contexts. Second, studies should incorporate various variables to enhance the explanatory power of the factors influencing nursing students’ nursing intentions regarding COVID-19 patients. Third, the survey instrument used in this study was recently developed, adapted, and modified to suit the study population and included vocabulary adjustments. Therefore, although an exploratory factor analysis was conducted, a confirmatory factor analysis was not performed. Fourth, future studies should develop and validate educational programs for nursing students that reduce their risk perceptions of novel infectious diseases, foster positive attitudes, increase preventive behavioral practices, and ultimately enhance nursing intentions in clinical settings.

## 5. Conclusions

This study provides a fundamental foundation that gives essential insights to strengthen systematic infection control measures and improve the quality of patient care, especially in the context of emerging infectious diseases such as COVID-19. This study made important connections by revealing that nursing students’ intentions toward patients with COVID-19 are intricately linked to their risk perceptions, attitudes, and prevention behaviors. The identified factors call for a targeted approach when educating nursing students, emphasizing correct infection control practices and the careful use of protective gear. Mitigating risk perceptions, fostering positive attitudes, and promoting adherence to preventive behaviors are emerging as pivotal aspects of creating a proactive and committed nursing workforce. Recognizing the paramount role of nursing students as frontline healthcare professionals after graduation, tailored training programs need to be devised. These programs should not only impart the necessary skills and knowledge, but also inculcate a sense of voluntary participation in nursing in clinical settings. Such initiatives aim to not only address existing knowledge gaps, but also to improve the overall quality of care provided by nursing professionals in the face of emerging infectious disease challenges. 

While we recognize the contributions of this study, it is important to approach its findings with an understanding of their nuances, given that there are numerous papers covering similar topics from 2021 to 2023. Carefully explaining the strengths and differences of our findings compared to existing research is critical to making a meaningful contribution to the existing body of knowledge. Future efforts should build on these foundational insights and work toward a more comprehensive understanding and validation of the associations identified among nursing students, their intentions, and the multifaceted dynamics of infectious disease care.

## Figures and Tables

**Table 1:** General characteristics of participants (*N* = 149).

Characteristics	Categories	*N* (%)
Sex	Male	16 (10.7)
	Female	133 (89.3)
Grade	1st year	36 (24.2)
	2nd year	33 (22.1)
	3rd year	30 (20.1)
	4th year	50 (33.6)
Religion	Yes	33 (22.1)
	No	116 (77.9)
Subjective health status	Poor	14 (9.4)
	Average	52 (34.9)
	Good	83 (55.7)
Clinical practice during the COVID-19 outbreak	Yes	64 (43.0)
	No	85 (57.0)

COVID-19 = Coronavirus Disease-19.

**Table 2:** Nursing students’ knowledge, trust in government guidelines, and perceived risk regarding COVID-19 (*N* = 149).

Knowledge	Correct Answer Rate (Range 0–100%)
COVID-19 transmission methods	97.3%
COVID-19 incubation period	91.3%
Initial symptoms of COVID-19	49.7%
Hand washing for COVID-19 prevention	95.3%
COVID-19 specimen collection location	40.9%
COVID-19 specimen collection method	26.2%
Wearing personal protective equipment (PPE) to prevent COVID-19 infection	83.9%
Management of the waste of COVID-19-confirmed patients	91.9%
Criteria for releasing isolation	51.7%
Total	69.8%
Attitudes; Trust in government guidelines	Mean ± SD
Trust in national control	3.44 ± 0.97
Trust in the nation’s resilience	3.81 ± 0.83
Trust in hospital guidelines and recommendations	3.98 ± 0.87
Total	3.74 ± 0.76
Perceived risk	Mean ± SD
Risk of infection from patient contact	3.62 ± 0.96
Risk of infection due to working in the isolation ward	3.91 ± 0.90
Risk of family transmission infection	4.42 ± 0.70
Total	3.98 ± 0.63

COVID-19 = Coronavirus Disease-19. SD = standard deviation.

**Table 3:** Preventive behaviors against COVID-19.

Items	Classification	Mean ± SD
1.	Reduced interaction with friends	1.33 ± 0.51
2.	Decreased use of public transportation	1.09 ± 0.64
3.	Avoidance of enclosed spaces	1.62 ± 0.51
4.	Practice of cough etiquette	1.87 ± 0.36
5.	Hygiene management for frequently touched objects	1.09 ± 0.68
6.	Reduced outdoor shopping	1.74 ± 0.47
7.	Increased frequency of hand washing	1.85 ± 0.39
8.	Avoidance of crowded areas	1.62 ± 0.50
9.	Increased mention of prevention	1.65 ± 0.52
10.	Increased use of alcohol swabs	1.48 ± 0.68
11.	Daily mask replacement	1.61 ± 0.52
12.	Minimization of family gatherings	1.67 ± 0.50
13.	Increased checking of disaster safety information	1.55 ± 0.58
Total		1.55 ± 0.29

COVID-19 = Coronavirus Disease-19. SD = standard deviation.

**Table 4:** Nursing intention (*N* = 149).

Classification	Mean ± SD
Active participation in the nursing care of COVID-19 patients	1.31 ± 1.34
Participation in the nursing care of COVID-19 patients when necessary	1.23 ± 1.44
Voluntary participation in the nursing care of COVID-19 patients	0.93 ± 1.64
Total	1.16 ± 1.40

COVID-19 = Coronavirus Disease-19. SD = standard deviation.

**Table 5:** Differences in nursing intentions for COVID-19 patients by general characteristics.

Characteristics	Categories	Nursing Intentions	
		Mean ± SD	t/F (*p*)
Sex	Male	1.81 ± 1.09	2.011 (0.046)
	Female	1.08 ± 1.41	
Grade	Freshman	0.94 ± 1.61	0.532 (0.661)
	Sophomore	1.13 ± 1.45	
	Junior	1.36 ± 1.45	
	Senior	1.21 ± 1.17	
Religion	Yes	1.04 ± 1.46	0.541 (0.589)
	No	1.19 ± 1.38	
Subjective health status	Poor ^a^	0.52 ± 1.94	4.144 (0.018) c > a
	Average ^b^	0.89 ± 1.49	
	Good ^c^	1.43 ± 1.16	
Clinical practice during the COVID-19 outbreak	Yes	1.14 ± 1.27	0.160 (0.873)
	No	1.17 ± 1.49	

COVID-19 = Coronavirus Disease-19. SD = standard deviation. ^a^, ^b^, ^c^ = Scheffe test.

**Table 6:** Correlation of variables.

Variables	Knowledge	Attitudes	Perceived Risk	Preventive Behaviors	Nursing Intentions
	r (*p*)	r (*p*)	r (*p*)	r (*p*)	r (*p*)
Knowledge	1				
Attitudes	0.12 (0.146)	1			
Perceived risk	0.08 (0.352)	−0.02 (0.789)	1		
Preventive behaviors	0.16 (0.030)	0.21 (0.010)	0.13 (0.117)	1	
Nursing intentions	0.03 (0.731)	0.34 (<0.001)	−0.36 (<0.001)	0.19 (0.020)	1

**Table 7:** Factors influencing nursing intentions for COVID-19 patients.

Variables	B	SE	β	t	*p*
(Constant)	1.16	0.88		2.49	0.014
Perceived risk	−0.84	0.16	−0.38	−5.31	<0.001
Attitudes	0.53	0.13	0.29	3.99	<0.001
Preventive behaviors	0.87	0.36	0.18	2.42	0.017
		R^2^ = 0.27, Adj. R^2^ = 0.26, F = 17.85, *p* < 0.001			

## Data Availability

The datasets analyzed during the current study are available from the corresponding author upon reasonable request due to privacy concerns.

## References

[1] Li Q., Guan X., Wu P., Wang X., Zhou L., Tong Y., Ren R., Leung K.S.M., Lau E.H.Y., Wong J.Y. (2020). Early transmission dynamics in Wuhan, China, of novel coronavirus–infected pneumonia. N. Engl. J. Med..

[2] Hong H.C., Lee H., Lee S.J., Park C., Lee M. (2023). The determinants of adherence to public health and social measures against COVID-19 among the general population in South Korea: National survey study. JMIR Public Health Surveill..

[3] Jackson D., Bradbury-Jones C., Baptiste D., Gelling L., Morin K., Neville S., Smith G.D. (2020). Life in the pandemic: Some reflections on nursing in the context of COVID-19. J. Clin. Nurs..

[4] Keles E., Bektemur G., Baydili K.N. (2021). COVID-19 deaths among nurses: A cross-sectional study. Occup. Med..

[5] Almaghrabi R.H., Alfaraidi H.A., Al Hebshi W.A., Albaadani M.M. (2020). Healthcare workers experience in dealing with coronavirus (COVID-19) pandemic. Saudi Med. J..

[6] Hua F., Qin D., Yan J., Zhao T., He H. (2020). COVID-19 related experience, knowledge, attitude, and behaviors among 2669 orthodontists, orthodontic residents, and nurses in China: A cross-sectional survey. Front. Med..

[7] Park S.J., Han J.E., Kwak K.H. (2021). The influence of nursing students’ knowledge, attitudes and infection prevention behaviors for COVID-19 upon the nursing intention for patients with the emerging infectious diseases. Korean Soc. Nurs. Res..

[8] Sun N., Wei L., Shi S., Jiao D., Song R., Ma L., Wang H., Wang C., Wang Z., You Y. (2020). A qualitative study on the psychological experience of caregivers of COVID-19 patients. Am. J. Infect. Control.

[9] Sun Y., Wang D., Han Z., Gao J., Zhu S., Zhang H. (2020). Disease prevention knowledge, anxiety, and professional identity during COVID-19 pandemic in nursing students in Zhengzhou, China. J. Korean Acad. Nurs..

[10] Gan W.H., Lim J.W., Koh D. (2020). Preventing intra-hospital infection and transmission of coronavirus disease 2019 in health-care workers. Saf. Health Work.

[11] Al-Hunaishi W., Hoe V.C., Chinna K. (2019). Factors associated with healthcare workers willingness to participate in disasters: A cross-sectional study in Sana’a, Yemen. BMJ Open.

[12] Liu Q., Luo D., Haase J.E., Guo Q., Wang X.Q., Liu S., Xia L., Liu Z., Yang J., Yang B.X. (2020). The experiences of health-care providers during the COVID-19 crisis in China: A qualitative study. Lancet Glob. Health.

[13] Jeong D., Eun Y. (2023). Factors influencing SARS-CoV-2 infection control practices of nurses caring for COVID-19 patients in South Korea: Based on health belief model. Int. J. Environ. Res. Public Health.

[14] Shim E., Tariq A., Choi W., Lee Y., Chowell G. (2020). Transmission potential and severity of COVID-19 in South Korea. Int. J. Infect. Dis..

[15] Cesare M., D’agostino F., Maurici M., Zega M., Zeffiro V., Cocchieri A. (2023). Standardized nursing diagnoses in a surgical hospital setting: A retrospective study based on electronic health data. SAGE Open Nurs..

[16] Mena-Tudela D., González-Chordá V.M., Andreu-Pejó L., Mouzo-Bellés V.M., Cervera-Gasch Á. (2021). Spanish nursing and medical students’ knowledge, confidence and willingness about COVID-19: A cross-sectional study. Nurse Educ. Today.

[17] Hung M.S.Y., Lam S.K.K., Chow M.C.M., Ng W.W.M., Pau O.K. (2021). The effectiveness of disaster education for undergraduate nursing students’ knowledge, willingness, and perceived ability: An evaluation study. Int. J. Environ. Res. Public Health.

[18] Lee M., Kang B.A., You M. (2021). Knowledge, attitudes, and practices (KAP) toward COVID-19: A cross-sectional study in South Korea. BMC Public Health.

[19] Kim H.R., Choi E.Y., Park S.Y., Kim E.A. (2020). Factors influencing preventive behavior against coronavirus disease (COVID-19) among medically inclined college students. J. Korean Acad. Fundam. Nurs..

[20] Taghrir M.H., Borazjani R., Shiraly R. (2020). COVID-19 and Iranian medical students; a survey on their related-knowledge, preventive behaviors and risk perception. Arch. Iran. Med..

[21] Park J.H., Chang S.J., Choi S. (2018). Correlation between knowledge, attitude, and compliance of preventive behaviors regarding Middle East respiratory syndrome among nursing students. J. Korean Biol. Nurs Sci..

[22] Gallè F., Sabella E.A., Da Molin G., De Giglio O., Caggiano G., Di Onofrio V., Ferracuti S., Montagna M.T., Liguori G., Orsi G.B. (2020). Understanding knowledge and behaviors related to COVID–19 epidemic in Italian undergraduate students: The EPICO study. Int. J. Environ. Res. Public Health.

[23] Park J.H., Kim J.H., Lee H.J., Kang P. (2021). The relationship of anxiety, risk perception, literacy, and compliance of preventive behaviors during COVID-19 pandemic in nursing students. J. Korean Soc. Appl. Sci. Technol..

[24] Luo J., Luo L., Yang A., Cui M., Ma H. (2023). Clinical experiences of final-year nursing students during the COVID-19 pandemic: A systematic review and meta-synthesis. Nurse Educ. Today.

[25] Kim H.J., Choi Y.H. (2016). Factors influencing clinical nurses’ nursing intention for high risk pathogen infected patient. J. Korean Clin. Nurs. Res..

[26] Kim J.S., Choi J.S. (2016). Factors predicting clinical nurses’ willingness to care for Ebola virus disease-infected patients: A cross-sectional, descriptive survey. Nurs. Health Sci..

[27] Kim H.S., Park J.H. (2018). Predictors of MERS-related preventive behaviors performance among clinical practice students in a tertiary hospital. J. Korea Acad. Ind..

[28] Zhong B.-L., Luo W., Li H.-M., Zhang Q.-Q., Liu X.-G., Li W.-T., Li Y. (2020). Knowledge, attitudes, and practices towards COVID-19 among Chinese residents during the rapid rise period of the COVID-19 outbreak: A quick online cross-sectional survey. Int. J. Biol. Sci..

[29] Peng Y., Pei C., Zheng Y., Wang J., Zhang K., Zheng Z., Zhu P. (2020). Knowledge, attitude and practice associated with COVID-19 among university students: A cross-sectional survey in China. BMC Public Health.

[30] Rana I.A., Bhatti S.S., Aslam A.B., Jamshed A., Ahmad J., Shah A.A. (2021). COVID-19 risk perception and coping mechanisms: Does gender make a difference?. Int. J. Disaster Risk Reduct..

[31] Lee J., Kang S.J. (2020). Factors influencing nurses’ intention to care for patients with emerging infectious diseases: Application of the theory of planned behavior. Nurs. Health Sci..

[32] Kim H., Cheon E.Y., Yoo J.H. (2021). A study on the relationship between knowledge, risk perception, preventive health behavior from coronavirus disease-2019 in: Nursing Students. J. Korea Acad. Ind. Coop. Soc..

[33] Hwang S., Lee M. (2020). Perceived knowledge, anxiety and compliance with preventive behavior performance on COVID-19 by nursing college students. J. Digit. Converg..

[34] Abdulrahman Yusuf K., Isa S.M., Al-Abdullah A.F., AlHakeem H.A. (2023). Assessment of knowledge, accessibility, and adherence to the use of personal protective equipment and standard preventive practices among healthcare workers during the COVID-19 pandemic. J. Public Health Res..

[35] Choi Y.E., Lee E.S. (2019). A Study on knowledge, attitude, infection management intention & educational needs of new respiratory infectious disease among nurses who unexperienced NRID (SARS & MERS). J. Korea Acad. Ind. Coop. Soc..

[36] Oyama N., Seki M., Nakai M., Miyamoto K., Nagao K., Morimitsu R. (2023). Depressive symptoms, burnout, resilience, and psychosocial support in healthcare workers during the COVID-19 pandemic: A nationwide study in Japan. PCN Rep..

[37] Park J.H., Chang S.J., Kim K.S. (2017). Correlation between the preventive behaviors on Middle East respiratory syndrome and the knowledge, attitude, and compliance of medically inclined college students. J. Dent. Hyg. Sci..

[38] Henderson A.A., Jeong S.S., Horan K.A. (2023). Trust in management and state government mitigate the relationships between individual-and state-level stressors and well-being during COVID-19. J. Manag. Psychol..

[39] Morgado-Toscano C., Gomez-Salgado J., García-Iglesias J.J., Fagundo-Rivera J., López-López D., Allande-Cussó R. (2023). Levels of anxiety and fear among nurses during the COVID-19 pandemic: A systematic review. J. Nurs. Manag..

[40] Kim J.Y. (2017). Nurses’ experience of Middle East respiratory syndrome patients care. J. Korea Acad. Ind. Coop. Soc..

[41] Lord H., Loveday C., Moxham L., Fernandez R. (2021). Effective communication is key to intensive care nurses’ willingness to provide nursing care amidst the COVID-19 pandemic. Intensive Crit Care Nurs..

[42] Kim O.S., Oh J.H., Lee K.H. (2016). The convergence study on anxiety, knowledge, infection possibility, preventive possibility and preventive behavior level of MERS in nursing students. J. Korea Converg. Soc..

[43] Cheung K., Chan C.K., Chang M.Y., Chu P.H., Fung W.F., Kwan K.C., Lau N.Y., Li W.K., Mak H.M. (2015). Predictors for compliance of standard precautions among nursing students. Am. J. Infect. Control.

[44] Park E., Park H.R., Lee J.H. (2023). Barriers to learning healthcare-associated infections prevention and control during clinical practicum among nursing students in Korea: A focus group study. Int. J. Environ. Res. Public Health..

[45] Moon H.J., Park J.Y. (2021). Factors influencing intentions to care for emerging infectious disease patients among national and public hospitals nurses. J. Korean Acad. Fundam. Nurs..

[46] Kim C.J., Yoo H.R., Yoo M.S., Kwon B.E., Hwang K.J. (2006). Attitude, beliefs, and intentions to care for SARS patients among Korean clinical nurses: An application of theory of planned behavior. J. Korean Acad. Nurs..

[47] Song M.S., Yang N.Y. (2021). Influence of nurses’ COVID-19 related stress, hardiness, and organizational citizenship behavior on nursing intention in infectious diseases hospitals. J. Korean Acad. Soc. Home Care Nurs..

[48] Jeong S.A., Kim J. (2021). Factors influencing nurses’ intention to care for patients with COVID-19: Focusing on positive psychological capital and nursing professionalism. PLoS ONE.

[49] Park D.K. (2020). The problems and lessons of emergency management in Korea: With the case of COVID-19. J. Korean Public Police Sec. Stud..

[50] Cowden J., Crane L., Lezotte D., Glover J., Nyquist A.C. (2010). Pre-pandemic planning survey of healthcare workers at a tertiary care Children’s Hospital: Ethical and workforce issues. Influenza Other Respir. Viruses.

[51] Seo H., Kim K. (2022). Factors influencing public health nurses’ ethical sensitivity during the pandemic. Nurs. Ethics.

[52] Jun J.J., Kim Y.H. (1996). Correlational study of health promoting life styles, self esteem and perceived health status of adulthood. J. Korean Acad. Soc. Adult Nurs..

[53] Choi J.S., Kim J.S. (2016). Factors influencing preventive behavior against Middle East Respiratory Syndrome-coronavirus among nursing students in South Korea. Nurse Educ. Today.

[54] Grimes D.E., Mendias E.P. (2010). Nurses intentions to respond to bioterrorism and other infectious disease emergencies. Nurs. Outlook.

